# Unusual anatomic variant of the axillary nerve challenging the deltopectoral approach to the shoulder: a case report

**DOI:** 10.1186/s13037-019-0189-1

**Published:** 2019-02-14

**Authors:** Richard A. Pizzo, Jeffrey Lynch, Donald M. Adams, Richard S. Yoon, Frank A. Liporace

**Affiliations:** 1 Division of Orthopaedic Trauma & Complex Adult Reconstruction, Department of Orthopaedic Surgery, Jersey City Medical Center – RWJBarnabas Health, 377 Jersey Ave, Suite 280A, Jersey City, NJ 07302 USA; 2 Rowan University School of Osteopathic Medicine, Stratford, NJ USA

**Keywords:** Axillary nerve, Reverse total shoulder arthroplasty, Anatomic variant

## Abstract

**Background:**

The deltopectoral approach is a well-described surgical approach to the proximal humerus and glenohumeral joint. One of the structures at risk during this approach is the axillary nerve. Typically, the axillary nerve arises off the posterior cord of the brachial plexus and courses lateral to the proximal humerus and inferior to the glenohumeral joint, exiting the axilla through the quadrangular space. We describe a case of an aberrant axillary nerve, coursing anteriorly across the glenohumeral joint within the deltopectoral groove encountered during a reverse total shoulder arthroplasty.

**Case presentation:**

A 73-year-old female presented complaining of atraumatic progressive right shoulder pain of several months duration. Clinical and radiographic findings were consistent with advanced rotator cuff arthropathy. After failing appropriate non-operative treatment, the patient elected to undergo reverse total shoulder arthroplasty. During the deltopectoral approach to the glenohumeral joint, the axillary nerve was found to be coursing deep to the cephalic vein within the deltopectoral interval. The nerve was isolated and protected, and the glenohumeral joint was accessed via a small window in the anterior deltoid muscle. The remainder of the procedure was performed without complication. The patient was found to be healing well and with normal axillary nerve function at 4-month follow-up.

**Conclusions:**

Neurologic lesions are well-documented complications of reverse total shoulder arthroplasty. The integrity of the axillary nerve is of particular importance to reverse total shoulder arthroplasty as it innervates the deltoid and post-operative function of the extremity is dependent upon a functioning deltoid muscle. Extreme care must be taken to avoid insult to the axillary nerve and any aberrant paths it may course around the glenohumeral joint.

## Background

The deltopectoral approach is a well-described and commonly utilized surgical approach to the glenohumeral joint and proximal humerus for both shoulder arthroplasty and fracture fixation [1–3]. The axillary nerve may be encountered in the inferior margin of this interval where it runs along the subcapularis prior to diving into the quadrangular space [4]. Laterally, it is traditionally located lateral to the proximal humerus, coursing from posterior to anterior around the proximal humerus [5]. Careful preservation of this nerve is critical, particularly in the setting of arthroplasty. Even so, neurologic lesions are relatively frequent and well-documented complications of reverse total shoulder arthroplasty [6]. Though many anatomic and cadaveric studies exist describing the location and course of the axillary nerve in this region, none have described an axillary nerve bypassing the quadrangular space and remaining anterior to the humerus within the deltopectoral interval to innervate the deltoid muscle [5, 7–9]. We describe a case of this anatomic variant that we encountered during the deltopectoral approach for reverse total shoulder arthroplasty.

## Case presentation

**Fig. 1:**
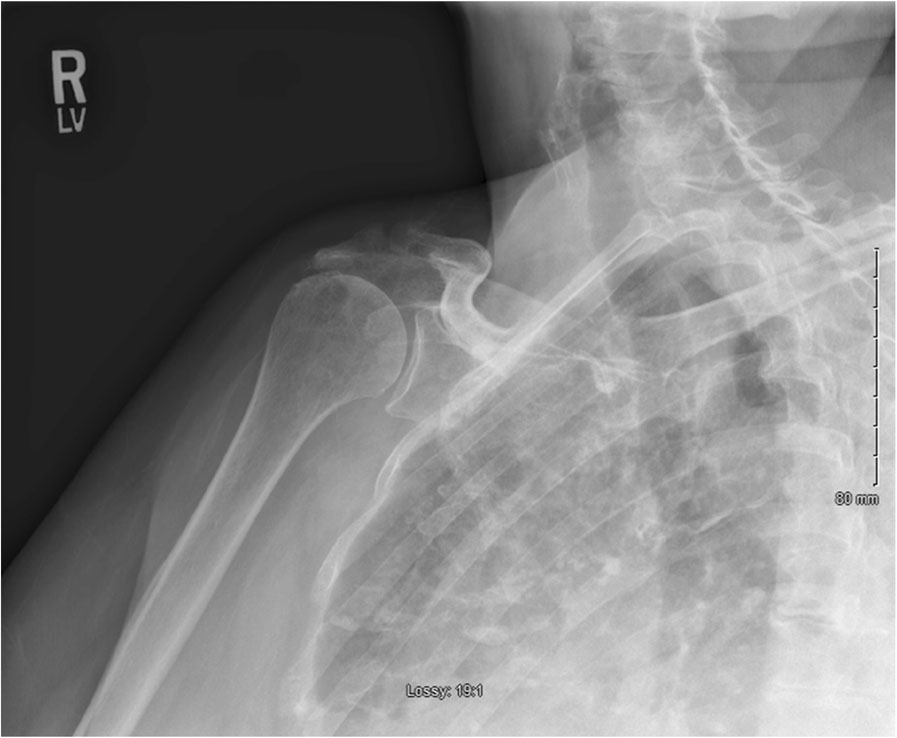
AP pre-operative radiograph of the right shoulder demonstrating superior migration of the humeral head and early acetabularization of the acromion

Patient is a 73 year-old right hand dominant female who initially presented to the office complaining of atraumatic right shoulder pain with activity and limited range of motion of longstanding duration. On physical exam, she was found to have significantly limited active range of motion of the right shoulder and clinical signs of impingement. Radiographs at that time demonstrated superior escape of the humeral head with impingement of the greater tuberosity on the acromion and early acetabularization of the acromion (Fig. 1). MRI findings were consistent with her x-ray and also demonstrated a lack of contiguous supraspinatus or infraspinatus tendon. At this juncture, the patient was diagnosed with rotator cuff arthropathy and elected to proceed with reverse total shoulder arthroplasty.

**Fig. 2:**
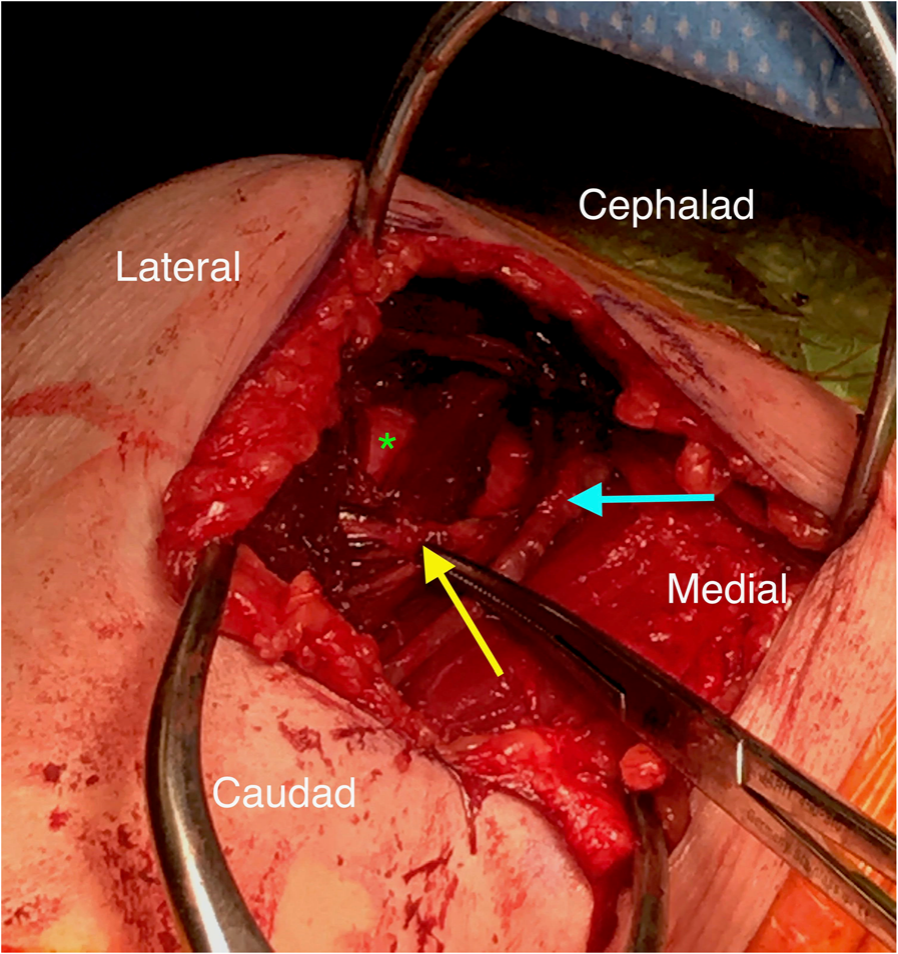
Intra-operative photograph via the deltopectoral approach to the glenohumeral joint. Yellow arrow: abberant axillary nerve, blue arrow: cephalic vein, green asterisk: humeral head

**Fig. 3:**
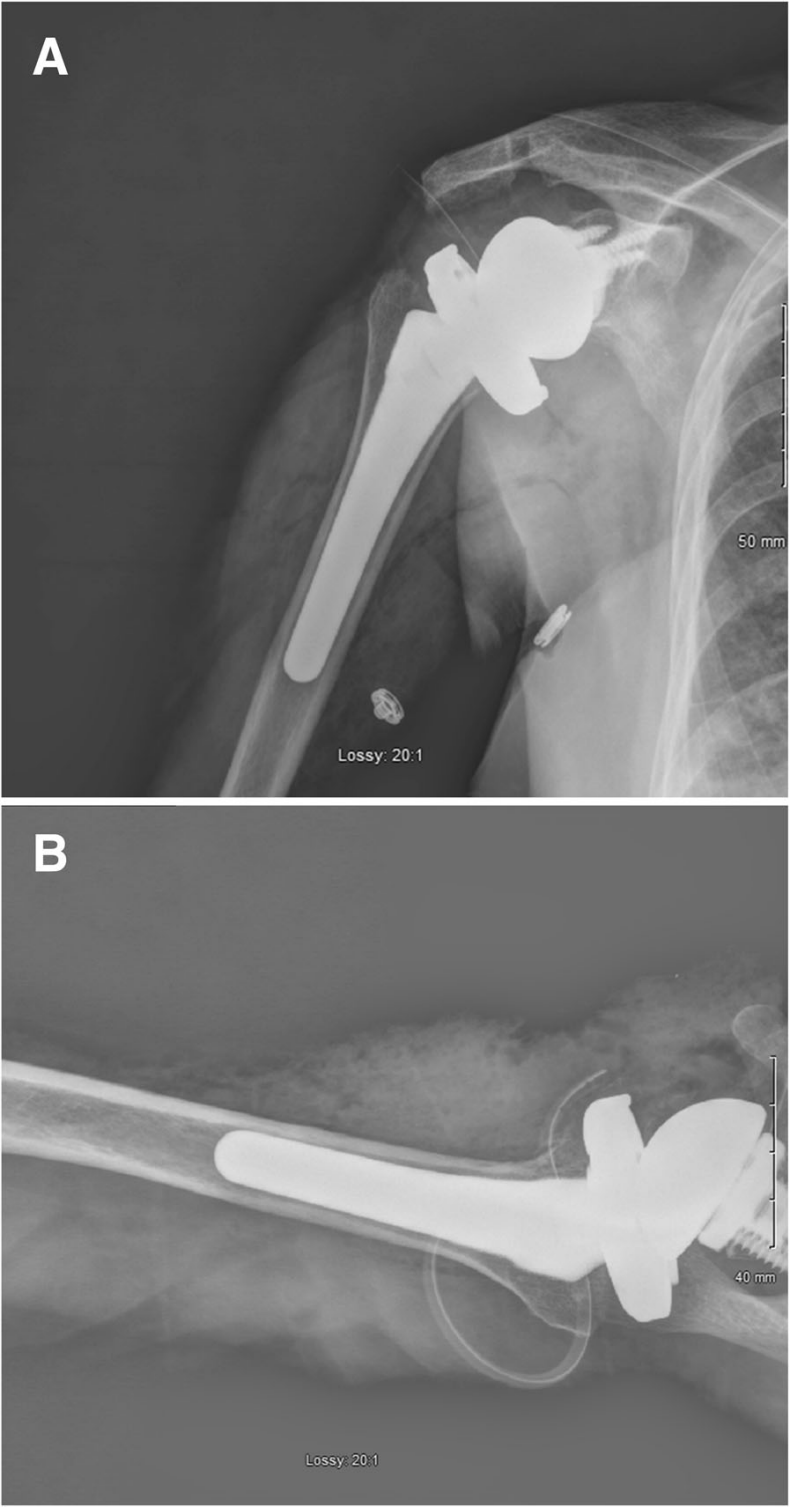
**a**-**b**: Post-operative radiographs demonstrating implant position and alignment

**Fig. 4:**
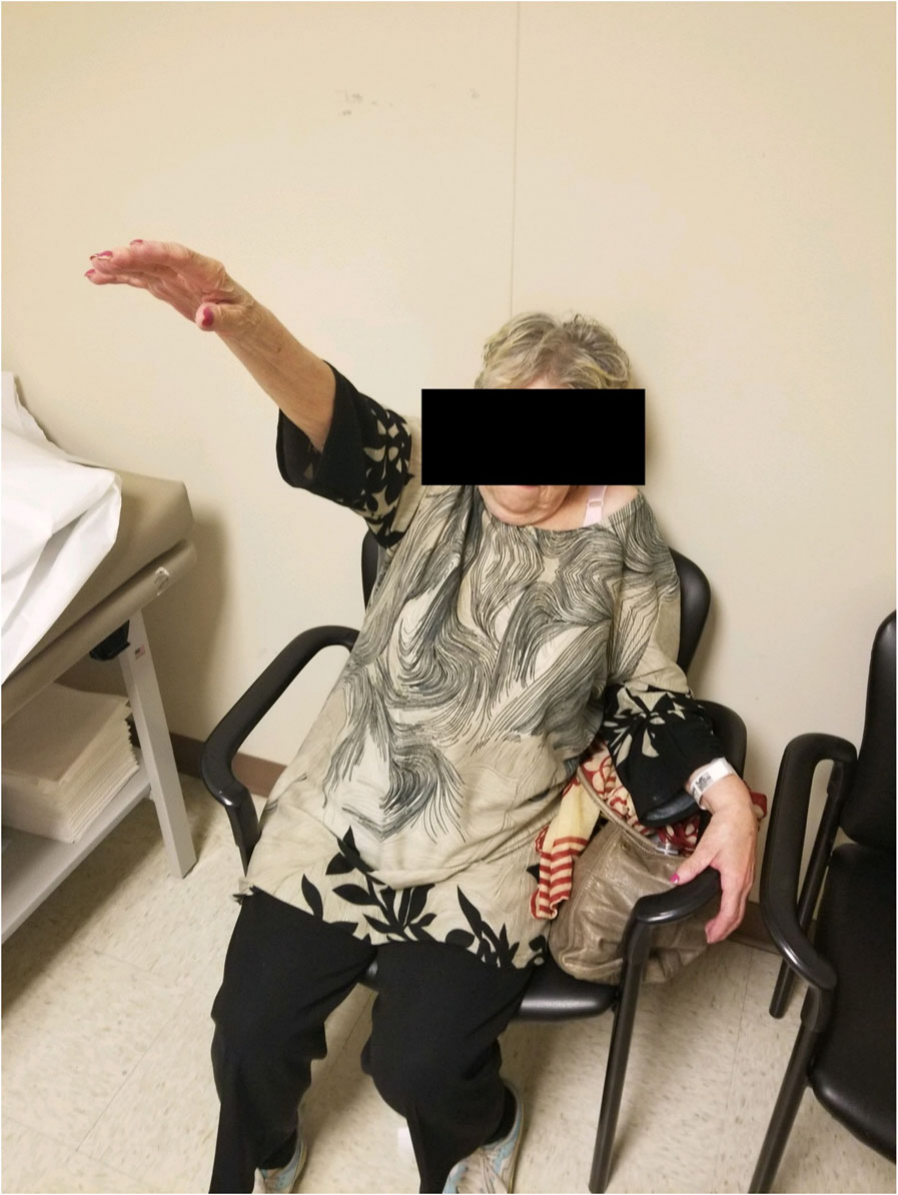
Clinical photo at 4-month follow-up demonstrating active forward flexion of shoulder to approximately 120 degrees

The patient was brought to the operating room and placed in the beach chair position. An incision was made from just lateral to the coracoid to the medial border of the proximal humeral shaft in line with the axillary fold. Subcutaneous tissue was dissected and the cephalic vein was identified. As the cephalic vein was mobilized and the clavipectoral fascia was incised, a discrete, branching, fascicular nerve was identified lateral and deep to the cephalic vein within the deltopectoral groove (Fig. 2). The nerve was further dissected and traced both proximally and distally. Distally, the nerve and all branches were found to be diving into the anterior deltoid muscle. Proximally, it was found to run deep to the conjoined tendon, towards the brachial plexus. The nerve was freed from the deltoid muscle belly, allowing enough excursion to access the glenohumeral joint via a small deltoid window. The remainder of the operation concluded without complication and the wound was closed primarily (Fig. 3). The patient was neurovascularly intact post-operatively with intact sensation in the axillary nerve distribution and able to fire her deltoid muscle. She healed without complications. At 4-month follow-up, she was doing well and able to actively abduct and forward flex her right shoulder to approximately 120 degrees (Fig. 4).

## Discussion

Neurologic lesions are well-documented complications of reverse total shoulder arthroplasty, occurring in up to 47% of patients [6]. While only 2% of these lesions are clinically significant and the majority of them fully resolve within 6 months, a permanent nerve injury can be disastrous [6, 10]. Integrity of the axillary nerve is of particular importance to reverse total shoulder arthroplasty, as one of the objectives of the procedure is to give mechanical control of the glenohumeral joint to the deltoid muscle [1–3]. If the axillary nerve is transected, vigorously retracted, or otherwise iatrogenically injured during the procedure, the patient may be left with a nonfunctional upper extremity. A thorough knowledge of the axillary nerve course and location is essential for patient safety and satisfaction.

The axillary nerve is one of two terminal branches of the posterior cord of the brachial plexus [8]. Typically, it travels from its origin to the inferior edge of the subscapularis muscle prior to diving into the quadrangular space and emerging posterior to the humerus and branching into the anterior and posterior branches [5, 8, 11]. The anterior branch wraps around the surgical neck of the proximal humerus in a posterior to anterior fashion approximately 5–7 cm from the lateral border of the acromion to supply the anterior portion of the deltoid muscle [7]. In the patient described, the axillary nerve exhibited multiple aberrancies. It was found to course lateral to the coracoid process instead of the described course 3.56 inferior to the coracoid [5]. The nerve also remained anterior to the subscapularis muscle belly, instead of traveling to the posterior aspect of the shoulder via the quadrangular space. Several cadaveric reports have described accounts of the axillary nerve not running through quadrangular space, however in these rare cases, the nerve either pierced the inferolateral subscapularis muscle or branched prior to entering the quadrangular space [4, 11]. There is one report of an axillary nerve coursing anterior to the humeral head and causing an irreducible shoulder dislocation, however this aberrant position is likely traumatic in nature [12]. The nerve described in this case continued on to supply the deltoid muscle from the anterior aspect of the humerus. Though variable innervation of different portions of the deltoid muscle by different axillary nerve branches have been described, the muscle is consistently supplied from branches that course posteriorly to the humeral head [11, 13–16].

## Conclusion

The axillary nerve provides motor innervation to the deltoid muscle, and its integrity is critical for patients undergoing reverse total shoulder arthroplasty. With such a high rate of neurologic complications and functional outcomes at stake, avoiding iatrogenic injuries to the axillary nerve is of paramount importance. We report here an important variant of the axillary nerve that ran within the deltopectoral interval anterior to the subscapularis muscle and coursing anterior to the humerus instead of diving into the quadrangular space. Identifying and reporting anatomic variants is essential to improving patient outcomes and avoiding iatrogenic injury.

## Abbreviations

MRI: Magnetic resonance imaging
